# Professional Competence and Spiritual Care Provision Among Zambian Maternal Healthcare Providers: Through the Lens of Watson's Theory of Human Caring

**DOI:** 10.1177/08980101251321970

**Published:** 2025-02-27

**Authors:** Atika Khalaf, Kalunga Cindy Nakazwe, Lena Halawi, Francis Sichimba

**Affiliations:** Faculty of Health Sciences, 4342 Kristianstad University, Kristianstad, Sweden; Hind Bint Maktoum College of Nursing and Midwifery, 472580 Mohammed Bin Rashid University of Medicine and Health Sciences, Dubai Health, Dubai, United Arab Emirates; Department of Psychology, 108234 University of Zambia, Lusaka, Zambia; Faculty of Health Sciences, 4342 Kristianstad University, Kristianstad, Sweden; Department of Psychology, 108234 University of Zambia, Lusaka, Zambia

**Keywords:** spiritual care competency, spiritual care provision, healthcare providers, maternal healthcare, Zambia

## Abstract

**Purpose:** Spiritual care is a multifaceted and integral part of holistic health within the medical standard of care, and it is a crucial component of healthcare providers’ (HCPs) job descriptions. This study aimed to investigate Zambian HCPs’ professional competence and practice of spiritual care in maternal healthcare settings, with a comparative focus on nurses. **Methods:** A cross-sectional design was applied among 311 maternal HCPs using an online survey with two validated instruments: Professional Competence in Spiritual Care (PCSC) and the Nurse Spiritual Care Therapeutics Scale (NSCTS). **Results:** Most participants were females (63.0%) and nurses or midwives (25.1% each). The mean PCSC score was 135.4 (SD = 26.5), indicating excellent competence in spiritual care. The mean NSCTS score was 24.5 (SD = 14.2), indicating that HCPs seldom provide spiritual care. No significant differences were found in PCSC scores across different HCPs (*p* = 0.065). However, midwives and medical practitioners scored significantly higher on NSCTS compared to nurses (*p* < 0.001). **Conclusions:** Zambian HCPs demonstrate excellent competence in spiritual care but seldom provide it. Nurses appear to provide inadequate spiritual care compared to other HCPs. These findings highlight the need for further investigation to identify barriers preventing nurses from delivering comprehensive spiritual care.

## Introduction

The nursing field has long incorporated various healing therapies in line with its holistic principles of healing the whole person (Thornton, 2019). Per the holistic principle, nurses and healthcare providers (HCPs) in general must address the body, mind, spirit, culture, socioeconomic background, and environment (Sessanna et al., 2021). The World Health Organization's (WHO) definition of health recognizes that holistic health is more than just the absence of disease and champions the need for HCPs to address the other facets of health, including the spiritual aspect of patient care (WHO, 2002). Thus, holistic nursing and holistic care recognize the interconnectedness of the body, mind, spirit, emotions, environment, relationships, and social and cultural aspects of life (Frisch & Rabinowitsch, 2019). One of the well-known holistic nursing theories is Watson's Theory of Human Caring (Watson, 2024) which emphasizes the importance of human connection, holistic care, and the nurturing of the patient's mind, body, and spirit. Central to this theory is the concept of “caritas,” which refers to a deep, altruistic love and the cultivation of a healing environment that supports the emotional and spiritual well-being of both the caregiver and the patient. This approach fosters a compassionate, empathetic relationship, promoting the idea that caring is a moral and ethical imperative in nursing practice. In the context of maternal healthcare, Watson's theory underscores the critical role of HCPs in delivering not only physical care but also addressing the spiritual needs of mothers.

Spiritual care is a multi-faceted aspect that is an integral part of holistic health (Li et al., 2022) as well as the medical standard of care (Lukovsky et al., 2021) and is a crucial component of an HCP's job description. Thus, practicing HCPs should be competent and confident in executing their skills and providing spiritual care safely and effectively. The body of research on the relationship between spiritual care and health shows that spiritual care significantly affects patients’ health and well-being (Farahani et al., 2019; Gijsberts et al., 2019), with better quality of life and increased patient satisfaction (Rego et al., 2020), improved health outcomes, including reduced pain sensitivity, stress, depressive tendencies, and the ability to manage illness's emotional and physical demands (Bahadorani et al., 2021). Further, spiritual care contributes to mental wellbeing as it has been shown to lower depression rates, improve patient acceptance of their condition and coping mechanisms (Vazifeh doust et al., 2020), and subsequently reduce hospitalization stays (Abu-El-Noor & Abu-El-Noor, 2021). However, it is still unclear how widespread the use of spiritual care is, especially in the context of Zambian maternal healthcare.

However, literature has shown that nurses frequently struggle to give spiritual care due to a variety of factors (Neathery et al., 2020). The fear of proselytizing, which is against ethical codes, is one factor, as is the belief that patient spirituality is private. Also, the provision of spiritual care can be overwhelming and time-consuming. Further, HCPs may lack knowledge and education about it; therefore, failing to recognize its significance and exacerbating the struggle by HCPs to offer spiritual care to patients (Mascio et al., 2022; Ronaldson et al., 2012). Additionally, HCPs adoption and usage of spiritual care could be affected by the lack of knowledge and poor attitudes (Baldacchino, 2006; Moosavi et al., 2019; Ramezani et al., 2014), prohibiting them from addressing spiritual concerns and making them think they are unable to provide spiritual care (McSherry & Jamieson, 2011). For example, nurses with heavy workloads frequently give patients physical health distress relief priority because they believe that providing spiritual care is time-consuming and has no immediate benefit (Baldacchino, 2006). In addition, many nurses claim that they lack sufficient institutional monitoring or assistance to properly provide spiritual care (McSherry & Jamieson, 2011).

Numerous other studies found that having a thorough knowledge of and a favorable attitude toward spiritual care are good indicators of its usage and implementation in clinical practice (de Diego-Cordero et al., 2022; Sezer & Ozturk Eyimaya, 2022; Syamsiah et al., 2020). For instance, Syamsiah et al. (2020) revealed that nurses’ behavior when providing spiritual nursing care can be influenced by their knowledge. Similarly, Azarsa et al. (2015) discovered that HCPs who have a more favorable attitude toward spiritual care provide their patients with more spiritual care. Other research has shown that HCPs who lack knowledge of spiritual care are less likely to use spiritual care (Farahani et al., 2019). Another study by Melhem et al. (2016) of Jordanian nurses found that those who had taken spiritual care courses tended to have favorable views of spirituality and spiritual care. In addition, possessing expertise in assessment and planning, intervention and evaluation, interpersonal spirituality, and intrapersonal spirituality are other essential tools in providing spiritual care (Jones et al., 2021; van Leeuwen et al., 2021). According to van Leeuwen et al. (2021), HCPs’ abilities for providing spiritual care included managing one's own opinions, addressing the problem, acquiring facts, talking about and planning spiritual care, offering and reviewing, and incorporating spiritual care into policy.

Although the significance of gathering accurate data on knowledge about spiritual care and the level of awareness among HCPs is acknowledged in the literature, there is a paucity of research regarding knowledge, attitudes, and practices of spiritual care among HCPs in Zambia. This is against the backdrop of research demonstrating that having sufficient knowledge about spiritual care is a potent tool for fostering optimistic attitudes and practicing spiritual care. The constitution of Zambia provides religious freedom, and the country has been formally recognized as a “Christian nation” with a (96%) Christian majority (Seale et al., 2022). Zambia being a Christian nation, preaching and prayer are allowed in hospitals. Like many other African countries, Zambian citizens consult with both biological and conventional as well as religious healers (Sichimba et al., 2022). Anecdotal evidence demonstrates prayers, the usage of anointing oil, and visits from religious organizations and church groups are permitted in Zambian hospitals. It is well-documented that religiosity inherently affects nursing practice (Taylor et al., 2014). Given that most patients are Christians and the context that promotes the practice of religion, it is essential for HCPs to provide spiritual care; yet it is unknown how much spiritual care they deliver to promote the patients’ overall health. The current study is expected to add insight to the ongoing discourse on spiritual care by adding data on Zambia regarding knowledge, perception, competence, and practice of spiritual care among HCPs, especially in maternal healthcare.

Watson's Theory of Human Caring provides a valuable framework for understanding the role of spiritual care in maternal healthcare settings, particularly in the context of Zambian nurses’ professional competence. This theory emphasizes the importance of holistic care that addresses not only physical needs but also emotional and spiritual aspects of patient well-being (Watson, 2013). In Zambia, where maternal healthcare faces significant challenges, the integration of spiritual care into nursing practice could potentially improve patient outcomes and satisfaction (Hanlon et al., 2014). Nurses, as primary caregivers, are uniquely positioned to provide spiritual support to pregnant women and new mothers, aligning with Watson's concept of transpersonal caring relationships (Watson, 2011). However, the extent to which Zambian maternal healthcare providers, especially nurses, incorporate spiritual care into their practice and how it relates to their professional competence remains understudied. This comparative focus on nurses within the Zambian maternal healthcare system offers insights into the potential benefits and challenges of implementing Watson's theory in a culturally specific context (Kim et al., 2020).

### Aim

This study aimed to investigate Zambian maternal HCPs’ professional competence, and practice of spiritual care in the maternal healthcare settings, with a comparative focus on nurses.

## Methods

### Study Design

This cross-sectional study was conducted among a convenience sample of HCPs across Zambia's 10 provinces from January to March 2024. Cross-sectional studies are commonly used in public health research to assess the prevalence of a condition or characteristic in a population at a specific point in time (Setia, 2016). Convenience sampling, a type of non-probability sampling, was employed due to the lack of a comprehensive sampling frame of all HCPs in Zambia (Elfil & Negida, 2017). While convenience sampling is subject to selection bias, it is often used when probability sampling is not feasible (Sharma, 2017). To ensure adequate representation, healthcare facilities of varying levels (primary, secondary, tertiary) and managing authorities (public, private, faith-based) were purposively included in each province (Etikan et al., 2016).

In addition, Watson's Theory of Human Caring (Watson, 2012, 2024; Yeter, 2015) guided this work. This theory emphasizes the importance of holistic care, which includes spiritual care, in nursing practice. It posits that caring is a fundamental aspect of nursing and that nurses should strive to create a caring environment that addresses patients’ physical, emotional, social, and spiritual needs (Sitzman & Watson, 2018).

### Sample and Setting

To ensure that the sample was diverse and representative of maternity healthcare in Zambia, the survey was sent to midwives, nurses, medical licentiates and obstetricians using a snowball technique to reach as many healthcare centers and hospitals across Zambia as possible. The main inclusion criteria were being clinically active and having encountered women during any phase of their perinatal journey (i.e. antenatal, delivery, and postnatal period). Hence, no specific exclusion criteria were applied. The sample size was calculated using an online sample size calculator (Calculator.net, 2012), following standard methodological guidelines. A 95% confidence interval and 5% margin of error were specified. Since the exact proportion of HCPs in the population was unknown, a conservative estimate of 50% was used as recommended when the population proportion is uncertain. The total population size of HCPs in Zambia was not available, so an unlimited population size was assumed, as is standard practice when the population size is unknown or very large in comparison to the sample. The calculated sample size was 385 individuals. However, the actual sample size, that is, those who responded to the survey, reached 311 HCPs.

Given the context of multiple religious natures, Zambia provides a unique context to study professional competency and the use of spiritual care in maternal healthcare settings as patients might have diverse religious beliefs and inclinations different from that of the provider.

### Instruments

Besides sociodemographic data (age, gender, profession, time in the profession, and time in the current unit), the following instruments were used.

#### Professional Competence in Spiritual Care

Professional Competence in Spiritual Care (PCSC) was designed to evaluate healthcare professionals’ abilities to provide holistic care that addresses patients’ spiritual needs alongside their physical and emotional well-being. It was developed based on a comprehensive literature review and expert opinions (Adib-Hajbaghery & Zehtabchi, 2016; Adib-Hajbaghery et al., 2017). The instrument consists of 32 items and multiple dimensions covering five key domains: assessment and implementation of spiritual care (17 items), human values (6 items), knowledge (4 items), attitudes (3 items), and self-recognition (2 items) (Adib-Hajbaghery et al., 2017). Scores range from 32 to 160. Achieving a total score of 118 or higher indicates excellent competence in spiritual care practice, while scores falling between 74 and 117 and below 73 are categorized as moderate and inadequate competence levels, respectively. The instrument's validity and reliability were established through rigorous testing, ensuring its effectiveness in measuring nurses’ competence in delivering spiritual care within healthcare settings, with a Cronbach's alpha of 0.93 for the total instrument. Permission to use the instrument was granted by the owner, Professor Adib-Hajbaghery, in written form (dated: October 8, 2023). The English version of the instrument was used.

This questionnaire was introduced as part B in the survey and assessed the participants’ knowledge, perception, competence, understanding and sensitivity toward the spiritual needs of patients and their families. For each item, participants were asked to indicate on a 5-point scale (1 = very low; 5= very much) the extent to which they agreed or disagreed with the items. Examples of items in part B were “I have good knowledge and understanding of various spiritual needs of patients and their families,” “I have the needed skills and ability to assess the spiritual needs of patients and their families,” and “I recognize the spiritual distress of a patient and his or her family.”

#### Nurse Spiritual Care Therapeutics Scale

The Nurse Spiritual Care Therapeutics Scale (NSCTS), developed by Mamier and Taylor (2015), is a robust instrument designed to measure the frequency of nurse-provided spiritual care the past three days, with five response alternatives stated as never (0 times), 1–2 times, 3–6 times, 7–11 times, and at least 12 times. Based on the sum of all items, high total scores suggest that HCPs frequently incorporate spiritual support to help patients integrate their spirituality, while low scores indicate that HCPs seldom provide spiritual care.

This 17-item scale demonstrates strong internal reliability, with an alpha coefficient of 0.93, and validity is supported by item-total correlations ranging from 0.40 to 0.80. Exploratory factor analysis revealed a single-factor structure with strong loadings, explaining 49.5% of the variance. The instrument's use is suggested not to be limited to a specific HCP group (Mamier & Taylor, 2015). The instrument, instructions, and permission to use it were granted through email contact with Professor Elisabeth Taylor, the owner of the instrument (dated: September 17, 2023).

Introduced as part C in the online survey, the NSCTS survey assessed participants’ use of spiritual care in their care provision during the past three days. For each item, participants were asked to select one of the response alternatives ordered from Never (0 times), to 1 to 2 times, 3 to 6 times, 7 to 11 times, and at least 12 times. Examples of items in part C were “Asked a patient about how you could support his or her spiritual or religious practices,” “Helped a patient to have quiet time or space for spiritual reflection or practices,” and “Listened actively for spiritual themes in a patient's story of illness.”

### Data Collection

Each healthcare setting was contacted by a researcher from the team. Potential participants were identified and recruited through healthcare facilities, and contacts known through the professional networks of the researchers. The recruited participants were sent the information sheet about the study and a link to the questionnaires through WhatsApp, short message services (SMS) and emails. The survey, conducted online using a trusted survey platform (Google Form), was sent to participants given a unique link to access the questionnaires. The recruited participants were also encouraged to share the link with other health professionals in their networks. The survey was open for 3 months (December, 2023 to February, 2024), and participants were free to complete the survey at their own pace.

The online questionnaire comprised three section parts. Section one was the information sheet, section two was the declaration of consent, and section three included the survey questions. The estimated time to complete the survey was between 10 and 15 minutes, as clearly stated in the information section.

### Data Analysis

The statistical package for SPSS version 29 was used to analyze the data. The data analysis was initiated by testing the quantitative variables for normal distribution (age, time in the profession, time in the current unit, total score of PCSC, and total score of NSCTS). Using Kolmogorov–Smirnov and Shapiro–Wilk tests (Krithikadatta, 2014), none of the tested variables except the total score of PCSC was normally distributed. Thus, non-parametric tests (Kruskal–Wallis’ *H*-test and Spearman's rho) were applied in conducting group comparisons and other inferential statistics. This was followed by a descriptive phase to summarize the demographic characteristics of the sample and the responses to the survey questions using frequencies, means, and standard deviations. Finally, an inferential analysis phase including *t*-tests and ANOVAs was employed.

For the dependent variable of PCSC, the total score was used in the further analyses; while for the non-normally distributed NSCTS, cut-off points were decided based on the distribution of the variable (quartiles); namely, excellent competence on a score of 32 or above, moderate on a score between 14 and 31, and inadequate competence on a score of 13 or below. A reliability test using Cronbach's alpha was run to test the internal consistency of the instruments in our multi-professional sample.

Some of the participants’ professional categories were merged to avoid low cell count leading to the formation of two categories, medical specialty and HCP. In the medical specialty category, the professions of medical licentiate, clinical officer, clinical medicine, and clinician were merged, while in the HCP category, those who identified themselves as HCPs, pharmacists, or laboratory persons were merged.

### Ethical Considerations

Ethical clearance from the Ethics Committee was granted to ensure the study's compliance with ethical standards. In addition, the first page of the survey contained the essential information about the study's aim, methods, and rationale and the contact information for the data collection responsible researcher in case of questions related to the study. Also, informed consent was obtained from all participants as a mandatory question to respond to access the survey. A clear explanation of the voluntary participation and the freedom to withdraw at any point without penalty was stated. Anonymity and confidentiality were maintained by coding responses although no personal data such as names, phone numbers, email addresses or identification numbers were collected.

## Results

A total of 311 HCPs, of which 37% were males and 63% females, participated in this study. Nurses and midwives formed the majority of the sample (25.1%, respectively), followed by medical practitioners (20.2%), medical specialty (19.3%), and other HCPs (10.3%). The total sample had a mean age of 23.8 years (SD = 5.3) with males slightly older than female participants (24.3 and 23.4, respectively). The mean time spent in the profession for all participants was 3.5 years (SD = 2.6), and 3.0 years in the current unit. Table 1 shows more details.

**Table 1.** Characteristics of the Participants

Variable	Total sample	Male *n* (%)	Female	PCSC		NSCTS	
				*m* (SD)	*p*-Value	*m* (SD)	*p*-Value
*Sex*					0.340^		0.760^
Male	115 (37.0)			136.5 (25.0)		25.0 (14.3)	
Female	196 (63.0)			134.8 (27.3)		24.2 (14.2)	
*Profession*					0.065^#^		**<0.001** ^#^
Nurse	78 (25.1)	11 (14.1)	67 (85.9)	133.6 (27.3)		19.2 (13.1)	
Midwife	78 (25.1)	26 (33.3)	52 (66.7)	141.5 (26.5)		29.2 (14.7)	
Medical practitioner	63 (20.2)	34 (54.0)	29 (46.0)	137.8 (26.2)		28.7 (13.6)	
Medical specialty	60 (19.3)	32 (53.3)	28 (46.7)	129.4 (23.2)		22.6 (12.1)	
Healthcare provider	32 (10.3)	12 (37.5)	20 (62.5)	131.4 (28.3)		21.2 (15.2)	
NSCTS, categorized					**<0.001^#^**		
Excellent	89 (28.6)	36 (40.4)	53 (59.6)	152 (24.9)			
Moderate	0	0	0	0			
Inadequate	222 (71.4)	79 (35.6)	143 (64.4)	128.7 (24.1)			
	***m* (SD) Min-Max**						
Age, years	23.8 (5.3) 18–43	24.3 (5.5) 18–41	23.4 (5.2) 18–43		0.547*		0.029*
Time in the profession	3.5 (2.6) 0–19	3.7 (2.3) 0.5–15	3.4 (2.8) 0.1–19		0.303*		0.034*
Time in the current unit	3.0 (1.9) 0–17	3.2 (1.6) 0.1–11	2.9 (2.1) 0.2–17		0.902*		0.681*
Total score of PCSC	135.4 (26.5) 62–190	136.5 (25.0) 76–190	134.8 (27.3) 62–190				
Total score of NSCTS	24.5 (14.2) 0–64	25.0 (14.3) 0–62	24.2 (14.2) 0–64				

The total sample frequencies are presented as column percentages while the comparison between males and females is presented in row percentages.

*Note.* PCSC= Professional Competency in Spiritual Care scale (score range= 38–190). NSCTS= Nurse Spiritual Care Therapeutics Scale (score range = 0–68). ^ Independent samples *t*-test. ^#^ Mann–Whitney *U*-test. * ANOVA.

The mean score of the PCSC scale for the whole sample was 135.4 (SD = 26.5) indicating excellent competence in spiritual care, and 24.5 (SD = 14.2) for the NSCTS indicating that HCPs seldom provide spiritual care, with no significant differences between males and females. Concerning the mean PCSC, no significant differences were found between the professions (*p*-value = 0.065). On the contrary, the mean score of NSCTS was significantly (*p*-value <0.001) higher among midwives and medical practitioners, 29.2 and 28.7, respectively, and lowest among nurses (19.2). Similarly, no significant differences were found in the PCSC regarding the participants’ age (*p*-value = 0.547), time spent in the profession (*p*-value = 0.303), and time in the current unit (*p*-value = 0.902). However, the NSCTS was significantly higher among the younger participants (*p*-value = 0.029), and the fewer years participants had served in their profession (*p*-value = 0.034) (Table 1).

Comparing the different professions pairwise showed no significant differences between professions except for between midwives and medical specialties (*t* = 2.80, *d* = 0.48, *p*-value = 0.006) with significantly higher PCSC mean scores among midwives (141.5 compared to 129.4 among medical specialties) (Table 1). The NSCTS on the other hand, showed significant mean score differences between nurses and midwives (*p*-value < 0.001), nurses and medical practitioners (*p*-value < 0.001), midwives and medical specialties (*p*-value = 0.006), midwives and HCPs (*p*-value = 0.012), medical practitioners and HCPs (*p*-value = 0.018), and medical practitioners and medical specialties (p-values= 0.009) (Tables 2). Spearman correlation analysis between the total scores of PCSC and NSCTS showed a moderate and significant correlation (correlation coefficient = 0.525, *p* < 0.001).

Further comparisons within the pairwise professions showed significant differences between male and female participants: nurses and midwives (*p*-value = 0.005), nurses and medical practitioners (*p*-value < 0.001), nurses and medical specialties (*p*-value < 0.001), nurses and HCPs (*p*-value = 0.006), midwives and medical practitioners (*p*-value = 0.014), and midwives and medical specialties (*p*-value = 0.018) (Table 2). The gender differences across professions regarding mean scores of PCSC and NSCTS are shown in Table 3.

**Table 2.** Cross-Professional Comparisons in Total Scores of PCSC and NSCTS (Independent Samples *T*-Test) and Sex (Pearson Chi-Square)

Cross-professional comparisons	PCSC			NSCTS			Male/Female
	*t*	*d* ^#^	*p*-Value*	*t*	*d* ^#^	*p*-Value*	*p*-Value^$^
RN – RM	−1.83	−0.30	0.069	−4.46	−0.71	**<0.001**	**0.005**
RN – MP	−0.91	−0.15	0.366	−4.20	−0.71	**<0.001**	**<0.001**
RN – MS	0.96	0.16	0.340	−1.54	−0.26	0.126	**<0.001**
RN – HCP	0.38	0.08	0.704	−0.68	−0.14	0.500	**0.006**
RM – MP	0.84	0.14	0.401	0.20	0.03	0.843	**0.014**
RM – MS	2.80	0.48	**0.006**	2.82	0.48	**0.006**	**0.018**
RM – HCP	1.78	0.37	0.078	2.57	0.54	**0.012**	0.676
**MP – HCP**	1.08	0.24	0.282	2.45	0.53	**0.016**	0.129
**MP – MS**	1.86	0.34	0.065	2.64	0.48	**0.009**	0.944
**HCP – MS**	0.37	0.08	0.716	−0.48	−0.11	0.631	0.148

*Note.* * *T*-test for equality of means, two-sided significance, equal variances assumed. **^#^** Cohen's *d* effect size. ^$^ Pearson Chi-square. RN = Registered Nurse, RM = Registered Midwife, MP = Medical Practitioner, MS = Medical Specialty, HCP = Healthcare Provider.

**Table 3.** Gender Differences Across Professions Regarding Mean Scores of PCSC and NSCT*S*

	PCSC, *m* (SD)		NSCTS, *m* (SD)	
Profession	Male	Female	Male	Female
RN	132.6 (32.4)	133.8 (26.7)	13.1 (8.2)	20.2 (13.6)
RM	143.6 (28.4)	140.5 (25.8)	29.9 (16.6)	28.8 (13.9)
MP	136.0 (24.5)	139.9 (28.5)	27.3 (12.5)	30.4 (14.7)
MS	131.2 (23.0)	127.5 (23.7)	23.5 (13.2)	21.5 (10.8)
HCP	140.6 (13.2)	125.9 (33.5)	22.8 (15.3)	20.3 (15.5)

*Note.* RN = Registered Nurse, RM = Registered Midwife, MP = Medical Practitioner, MS = Medical Specialty, HCP = Other Healthcare Provider.

The internal consistency of the instruments was tested using Cronbach's alpha. The reliability tests showed a Cronbach's alpha of 0.948 and 0.954 for the PCSC and NSCTS scales, respectively, indicating a high level of internal consistency for the scales with this specific multi-professional sample.

## Discussion

Providing spiritual care as an HCP and assessing the spiritual needs of patients and their families is a significant part of an HCP's role, especially that of a nurse. Given the scarcity of research regarding knowledge, attitudes, and practices of spiritual care among nurses in Zambia, and obscurity regarding the extent of spiritual care offered by HCPs, especially nurses in a Zambian context, this study was carried out. The study aimed to examine the competence of a multi-professional sample of Zambian HCPs in providing spiritual care, with a comparative focus on nurses. We found that the mean PCSC score among HCPs indicated excellent competence in spiritual care, while the mean NSCTS score was low indicating that HCPs seldom provide spiritual care. Furthermore, the findings suggest that age and time in the profession were significantly associated with higher frequencies of spiritual care provision. In addition, no significant differences were found in PCSC scores across different HCPs. However, midwives and medical practitioners scored significantly higher on NSCTS compared to nurses.

In the context of empirical research, there is an existing consensus regarding the importance of spiritual care and its necessity in upholding an individual's health and well-being, by nurturing and attaining spiritual well-being (Balboni et al., 2022). Our results showed that Zambian HCPs possessed excellent competence in spiritual care, but a low level of spiritual care provision, especially among nurses. This aligns with prior research suggesting that HCPs, especially nurses, tend to refrain from providing spiritual care to their patients and assessing patients’ spiritual needs; hence, the frequency of performance of spiritual care is deficient (Armitage, 2023). Our findings can be seen as fortifying a deficient practice of the use of spiritual care among nurses in a Zambian context compared to other HCPs (Bolarinwa et al., 2023). In this regard, our findings suggest that there is a strong indication of a difference in the level of spiritual care provision attributed to profession type which calls for further training and education on spiritual care for nurses and other HCPs to bridge the gap and ensure comprehensive and holistic care. Furthermore, the study's findings on the NSCTS highlight the importance of caring behaviors in providing spiritual care which is in line with Watson's Theory of Human Caring (Watson, 2024). The theory suggests that nurses should engage in behaviors that promote spiritual well-being, such as listening, empathy, and compassion (Safaan et al., 2024).

Furthermore, our findings suggest that younger HCPs have a higher frequency of providing spiritual care. This is consistent with previous research delineating that the provision of spiritual care is influenced by age, with younger nurses being more willing to provide spiritual care (DeKoninck et al., 2016; Green et al., 2020), indicating that age plays a crucial role in shaping nurses’ attitudes and practices toward spiritual care (Green et al., 2020; Tuck et al., 2001). Although our findings add to the existing evidence, there is a need for further research on the relationship between age and the provision of spiritual care. Additionally, the findings suggest that the frequency of spiritual care provision among HCPs may not directly relate to their years of experience in the profession. Instead, the caring environment and the approach to healthcare may play a more significant role in determining the frequency of spiritual care provision. This aligns with Watson's Theory of Human Caring, which emphasizes the importance of fostering a caring-healing environment and promoting a holistic approach to healthcare that includes spiritual care (Wei & Watson, 2019). To improve the frequency and quality of spiritual care provision in the Zambian maternal healthcare context, healthcare organizations are recommended to focus on creating a uniform caring environment that supports the spiritual needs of patients and their families. This can be achieved by providing spiritual care training (Hoffert et al., 2007), fostering a culture of compassion (Rachel et al., 2019), and incorporating spiritual care into daily practice (Connerton & Moe, 2018).

In relation to the perceived professional competence of providing spiritual care, our findings indicate a favorable competency in spiritual care practice in Zambia among HCPs, specifically among midwives, possessing the highest level of competence compared to medical practitioners and nurses. However, nurses’ level of competency in spiritual care was favorable, which is inconsistent with previous studies that have shown a moderate level of competency among nurses (Ebrahimi et al., 2017; Sabsevari et al., 2013). This suggests that nurses’ competency, encompassing their knowledge, skills, and abilities in providing spiritual care, is above average in the Zambian maternal healthcare context compared to other settings. This is expected, given the religious and spiritual values that persist in the social context of Zambia (Austad et al., 2023). Our findings are consistent with previous studies examining the perceptions of spiritual care in disparate cultures and reporting satisfactory comprehension of spiritual care among nurses, as well as readiness (Cetinkaya et al., 2013; Guo et al., 2024; Wong et al., 2008; Zakaria Kiaei et al., 2015). While there is a favorable level of competency, that is knowledge, skill, and ability to provide spiritual care among nurses, the actual provision was shown to be deficient in our study, suggesting a need to improve the facilitation and promotion of spiritual care among nurses in Zambia. In this regard, it is worth noting that literature has shown there is a high patient versus nurse ratio in Zambia (Tende et al., 2022). Thus, it is plausible that the high patient numbers and increased workload negatively impact nurses and potentially contribute to the lack of spiritual care provision among nurses in Zambia, which underscores the need for further research and elucidation on this matter (Zhang et al., 2023). Furthermore, an acceptable patient-to-nurse ratio could enhance the transpersonal caring process (Watson, 2024), which involves the nurse's ability to connect with the patient on a spiritual level, fostering a sense of unity and understanding (Nunes et al., 2020). Other studies have fortified the lack of actual provision of spiritual care among nurses in other social contexts, reporting a small number of nurses providing spiritual care (Monir et al., 2009; Wong & Yau, 2010). This was attributed to various challenges such as time constraints, a lack of knowledge, a failure to recognize spiritual care's significance and a lack of education as considerable barriers standing in the way of the actual provision of spiritual care (Green et al., 2020; Zhang et al., 2023). However, with the use of the PSCS instrument, our findings do not support the presence of failure to recognize the significance of spiritual care and a lack of knowledge as potential barriers regarding the lack of provision of spiritual care among nurses (Bolarinwa et al., 2023). Instead, it may be that there is a lack of education regarding how to best implement spiritual care, which can potentially be linked to time constraints, which can be seen as an umbrella term that constitutes constraints concerned with workload management and resource allocation that could potentially be deficient and scarce in the clinical settings of nurses, which is proved in previous research (Ebrahimi et al., 2017; McSherry & Jamieson, 2011; Momeni et al., 2022; Thomas, 2022). One of the essential components of Watson's theory of human caring is the transpersonal caring-healing part (Nunes et al., 2020) which emphasizes the HCP's intentional focus on providing care, thus enhancing their healing presence. This theory also acknowledges the therapeutic benefits of transpersonal connections and underscores the importance of delivering care that addresses the whole person, encompassing physical, emotional, and spiritual dimensions (Watson, 2024).

Another significant contribution of our study is the findings contradicting the existing literature that has primarily identified lack of knowledge and poor attitudes as the primary barriers hindering the use of spiritual care (Baldacchino, 2006; Green et al., 2020; Moosavi et al., 2019; Ramezani et al., 2014). The lack of knowledge and skills regarding spiritual care among nurses is not supported, given the favorable level of competency among nurses in our study. The reasons for our findings are suggested to be a result of environmental factors such as lack of medical equipment and poor staffing (Micheal & Drateru, 2019) and cultural aspects such as religious and cultural practices (Buser et al., 2020) that might influence the relationship between nurses and their patients (Anshasi et al., 2024), which warrants further research and elucidation on the matter, especially in a Zambian context.

Albeit spiritual health is the path toward inner peace regardless of the turmoil one is going through as a patient and should be upheld by healthcare systems and HCPs, especially nurses (Fradelos et al., 2016). As a human being, one is not merely a body, but a mind, body, and spirit. Knowing what spiritual care entails, namely possessing the knowledge, skills, and ability to provide it to patients, is one aspect. However, being able to effectively implement it is another, which is reinforced by our findings.

### Implications for Education and Practice

The practice of holistic nursing and healthcare involves a level of education as well as training for the attainment of proficiency within the field, but the efficiency of education and training is only as good as the implementation of the knowledge gained. In this regard, our study implies the necessity of continued professional development among nurses to advance practice in holistic nursing and healthcare, due to the lack of unification between theory and practice. In other words, there is a need for reconciliation between knowing and acting upon one's knowledge in practice of holistic nursing and healthcare with regard to spiritual care. While competence in spiritual care exists among nurse practitioners, its existence is in theory, not implemented practically, which implies the significance of strengthening nurses’ abilities to actually be able to provide spiritual care that falls in line with being a holistic part of nursing practice. Hence, our study implies a need for reconciliation between the ability to know and act as a nurse in the provision of spiritual care through a consistent process of learning and growing, allowing for what has been learnt about spiritual care to better become part of the behavior of nurses and holistic nursing practice. This requires the facilitation of a uniform caring environment that dismantles what can be perceived as a ‘check the box’ culture and adopting a culture that nurtures the maintenance and growth of skills among nurses. This may be accomplished through inter-professional training, workshops, and in-service education that promote experiential learning opportunities that elicit reflection among nurses regarding their encounter with patients. It is referred to as reflective observation, eliciting one to draw a conclusion from the experience through abstract conceptualization. It enables one to draw a conclusion from their experience and enables one to think, analyze, and plan the next steps forward in patient encounters to come, resulting in the application of what is learned, which is equivalent to active experimentation (Van Den Bossche & Baktrian, 2021). Hence, clinical encounters with patients should stimulate continuous learning among nurses, as well as comprehension of their profession and what it means to provide/practice holistic nursing. It should aid and elicit the construct of their professional identity and continued development as holistic nurses for the advancement of knowledge and practice of holistic nursing. This can potentially allow the knowledge gained by nursing practitioners to be put to work in a manner that the skills become a habit, a holistic habit for all involved in the reciprocal nurse-patient relationship.

### Implications for Research

On the subject of healing, holistic nursing practice underscores the reciprocalness of the mind, body, and spirit, which necessitates a whole-person care approach that considers all dimensions of health, namely, physical, emotional, social and spiritual dimensions. Our study verifies the significance of spiritual care as an essential aspect of holistic nursing care and inclusion as part of nurses’ provision of therapeutic interventions. Concurrently, our study affirms that there is an insufficient provision of spiritual care by nurses compared to other HCPs. Our findings suggest that being competent in spiritual care, as measured by knowledge, skills, and ability, does not necessarily translate to the actual provision of spiritual care in healthcare settings, particularly among nurses. In other words, competency does not guarantee performance, and the underlying reasons for this disparity remain unclear, warranting further advancement of research into the matter. Hence, based on our findings, we strongly recommend the implementation of efficient strategies to reinforce the use of spiritual care in healthcare provision, particularly among nurses. This necessitates further research to elucidate the primary barriers contributing to the lack of spiritual care provision among nurses in Zambia by relaying the voices of those faced with these barriers daily to improve upon the provision of holistic nursing and healthcare. Identifying these barriers is crucial to develop targeted interventions and examine their efficiency in counteracting the obstacles hindering the implementation of spiritual care, as well as practice of holistic nursing and healthcare.

In addition, to enhance in-depth understanding, we recommend that future research incorporate qualitative methods, such as semi-structured interviews or focus groups. These approaches would facilitate richer data collection by capturing participants’ voices and experiences more authentically. Qualitative methods can provide nuanced insights into complex phenomena, complementing quantitative findings and offering a more comprehensive understanding of the employed spiritual care practices. This mixed-methods approach would address potential limitations of solely quantitative data collection, allowing for a more holistic exploration of humanized and person-centered care and spiritual professional competencies in maternal healthcare settings.

### Strengths and Limitations

One of the methodological strengths of our study is the use of the validated PSCS and NSCTS instruments to examine not just nurses’ perceived competence in spiritual care but all kinds of HCPs’ perceptions of competency. It renders the instruments reliable in a multi-professional sample, which can be considered a crucial hallmark of this study. These instrumental tools serve as valuable resources for assessing and enhancing HCPs’, especially nurses’ proficiency in addressing the spiritual aspects of patient care, contributing to improved patient outcomes and overall quality of care in the Zambian healthcare context.

Despite our findings, there are some limitations to be considered. There was an uneven gender distribution comprising our study sample, with more females (63%) than males (37%) participating in this study. Although there were no significant differences between genders in this study, literature has delineated that for instance, female and male nurses differ in their perception of spirituality and spiritual care (Abusafia et al., 2021; de Diego-Cordero et al., 2022). In this regard, distinctions between female and male participants can lead to substantial scale score floor and ceiling effects and should be considered in future studies. The reason is, that floor- and ceiling effects can impact the capability of the instrument used to detect an increase or a decrease (Cramer & Howitt, 2004) in spiritual care competency among respondents who have high or low degrees of competency. Therefore, it is essential to differentiate between participants who have high or low spiritual care competency. Therefore, a sample with a balanced gender distribution can be seen as essential in distinguishing a gender difference in the assessment of spiritual care competency, in future studies. Secondly, inferences made apply exclusively to the study sample, which means that the inferences cannot be generalized to all HCPs in Zambia, which is due to raw summed total scores being item and sample-dependent (Hobart & Cano, 2009; Hobart et al., 2004). Despite the limitations of this study, it can be seen as a step forward in initiating interest and contributing to the literature on the topic of spiritual care in the Zambian context. The findings of this study could be used to increase acknowledgement and consideration of spiritual care by policymakers, leading to the development of policies and goals that enable better environmental factors for all healthcare personnel, especially nurses, to provide spiritual care to patients as needed. However, in this regard, this study recommends the implementation of qualitative research to specifically clarify the existing barriers experienced by nurses that hinder the actual provision of spiritual care in the Zambian context.

Furthermore, one of the inclusion criteria stipulated that HCPs should be clinically active and have encountered women during at least one phase of the peripartum period (i.e. prenatal, delivery, or postpartum). However, a potential confounding variable that warrants consideration is whether HCPs’ previous experiences with patient loss or bereavement influence their sensitivity toward providing compassionate and holistic care. This study did not explicitly address this factor, which represents a limitation. Future research should consider investigating the relationship between HCPs’ exposure to patient loss and their approach to care delivery, particularly in terms of spiritual and emotional support.

## Conclusions

Our study findings indicate that while practicing maternal HCPs in a Zambian context seem competent and confident in executing their skills in spiritual care, they seldom provide spiritual care and support to their patients. In addition, the extent to which nurses provide spiritual care to patients is deficient compared to other health providers, which posits the need for further inquiry into the matter. Additionally, it warrants the need to elucidate the barriers to better know how to counteract the hindrances posed by said barriers.

The spiritual aspect of patient care is crucial and warrants increased advocacy for implementation in clinical practice. To facilitate this goal, a growing body of research offers guidance for the promotion and development of the implementation of spiritual care.
